# The Biophysics of Flash Radiotherapy: Tools for Measuring Tumor and Normal Tissues Microenvironment

**DOI:** 10.3390/antiox14080899

**Published:** 2025-07-23

**Authors:** Islam G. Ali, Issam El Naqa

**Affiliations:** 1 Department of Physics, Faculty of Science, Arish University, Arish 45511, Egypt; islam.gamal@sci.aru.edu.eg; 2 Departments of Machine Learning and Radiation Oncology, Moffitt Cancer Center, Tampa, FL 33612, USA

**Keywords:** flash radiotherapy, biophysics, microenvironment, tumor response, normal tissue protection, computational modeling, experimental systems, in vivo imaging

## Abstract

Ultra-high dose rate radiotherapy known as Flash radiotherapy (FLASH-RT) offers tremendous opportunities to improve the therapeutic ratio of radiotherapy by sparing the normal tissue while maintaining similar tumoricidal efficacy. However, the underlying biophysical basis of the FLASH effect remains under active investigation with several proposed mechanisms involving oxygen depletion, altered free-radical chemistry, and differential biological responses. This article provides an overview of available experimental and computational tools that can be utilized to probe the tumor and normal tissue microenvironment. We analyze in vitro, ex vivo, and in vivo systems used to study FLASH responses. We describe various computational and imaging technologies that can potentially aid in understanding the biophysics of FLASH-RT and lead to safer clinical translational.

## 1. Introduction

The therapeutic application of ionizing radiation in cancer management has evolved significantly in the past century. Conventional radiotherapy (CONV-RT), typically delivered in small daily fractions of 1.8–2.0 Gy/fraction at dose rates on the order of ∼0.3 Gy/s over several weeks, has demonstrated remarkable efficacy in tumor control but is limited by dose-related toxicities to surrounding healthy tissues [1]. As precision in radiation delivery and understanding of radiobiological processes improved, novel modalities are being investigated to enhance the therapeutic index. One such recent innovation is FLASH radiotherapy (FLASH-RT), a novel modality in which therapeutic radiation doses are delivered at ultra-high dose rates (UHDR)—typically ≥40 Gy/s—over very short irradiation times [2,3,4]. This modality was first shown in 2014 to markedly spare normal lung tissue in mice while maintaining tumor control [5], and subsequent work has confirmed the so-called the “FLASH effect” of normal-tissue protection in multiple organs (e.g., lung, intestine, brain, skin, etc.) without loss of antitumor efficacy [6]. This sparing of healthy tissue—the FLASH effect—represents a potential paradigm shift, broadening the therapeutic window of radiation therapy [3,7]. The underlying biophysical basis remains under active investigation, with several proposed mechanisms involving oxygen depletion, altered free-radical chemistry, and differential biological responses (e.g., immune and vascular effects) [8,9].

While prior reviews have explored isolated facets of the FLASH effect—from oxygen depletion and radical recombination models to early clinical case reports—this article represents the first comprehensive, methodology-driven critique of FLASH-RT biophysics. We integrate computational simulations, real-time sensor platforms, and organ-on-chip systems within a single framework to map each tool onto specific microenvironmental metrics, from fiber-optic oxygen probes and photoacoustic pO_2_ mapping to Monte Carlo track-structure and multi-scale stochastic modeling. By systematically reviewing in vitro, ex vivo, and in vivo studies, we identify critical gaps in measurement capabilities—such as the need for physiologically relevant oximetry—and propose a coordinated roadmap for leveraging emerging technologies to refine mechanistic models, optimize treatment planning algorithms (including oxygen-depletion and free-radical kinetics), and accelerate safe, efficacious clinical translation of FLASH-RT.

## 2. Biophysics of FLASH-RT

**Figure 1:**
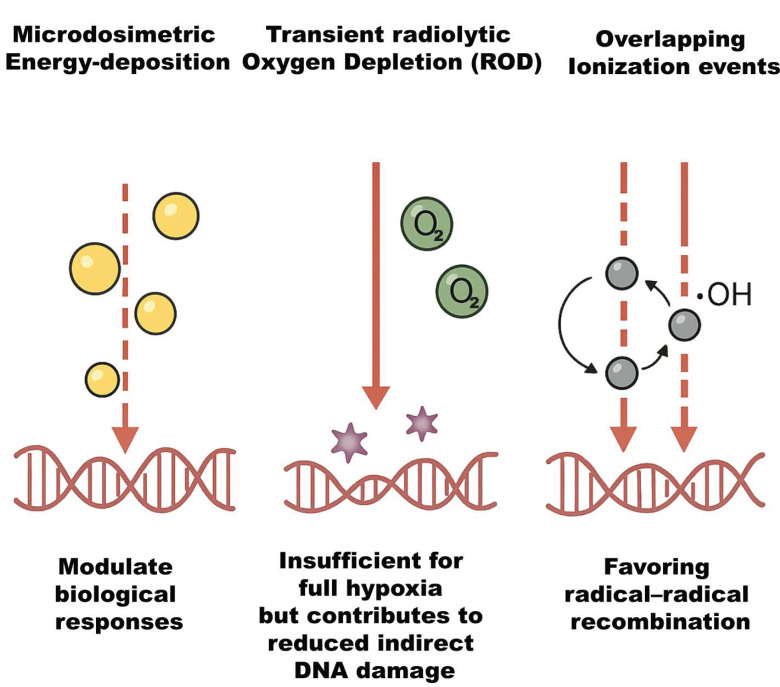
Schematic representation of key biophysical phenomena influencing radiobiological outcomes. The illustration highlights the distinct microdosimetric energy-deposition patterns that modulate cellular responses, transient radiolytic oxygen depletion (ROD) that, while insufficient to induce full hypoxia, reduces indirect DNA damage, and overlapping ionization events that enhance radical–radical recombination, thereby altering the balance of chemical species involved in radiation-induced damage.

With its UHDR pulses spread within milliseconds or less, FLASH-RT is a significant departure from CONV-RT [8]. The extreme dose rate has important implications. First, all radiation (and thus most DNA damage) occurs within milliseconds, effectively a single fraction without time for sublethal damage repair during irradiation. Secondly, because normal tissue sparing occurs without loss of tumor kill, the therapeutic index appears to improve [10]. The early physicochemical processes of radiation interaction, such as radical recombination, oxygen chemistry, and ionization event creation, are significantly altered by this high dose rate, resulting in less net oxidative damage in healthy tissues [4]. Important biophysical phenomena, as illustrated in Figure 1, include distinct microdosimetric energy-deposition patterns that modulate biological responses, transient radiolytic oxygen depletion (ROD) that is insufficient for full hypoxia but contributes to reduced indirect damage, and overlapping ionization events that favor radical–radical recombination [11]. Optimizing FLASH methods, dosimetry, and eventually clinical translation require understanding these processes.

### 2.1. Dosimetric and Practical Considerations

Implementing FLASH-RT clinically requires precise dosimetry to ensure appropriate UHDR delivery while managing beam characteristics. Physical measurements confirm that conventional linear accelerators (LINACs) can be adapted to FLASH by increasing pulse charge and minimizing spill-over, but verifying dose uniformity and real-time monitoring remain impending challenges [12]. The radiobiological mechanisms driving the FLASH effect remain incompletely understood, necessitating advanced dosimetric methods to resolve energy deposition at microscopic scales. Microdosimetry, which quantifies stochastic energy deposition in cellular or subcellular volumes, is pivotal for elucidating the spatial and temporal patterns of radiation interactions that underpin FLASH-specific biological outcomes [13,14]. Microdosimetric studies using Geant4-DNA demonstrate that FLASH pulses produce dense clusters of energy deposition over sub-micron scales, altering the spatial distribution of ionization and favoring localized radical recombination [15].

Conventional dosimetry protocols, optimized for standard dose rates, face significant limitations under FLASH conditions. Ionization chambers, for instance, exhibit substantial recombination losses in ultra-high dose-per-pulse regimes, necessitating corrections that introduce uncertainties [16,17]. Passive detectors such as radiochromic films (e.g., Gafchromic EBT3) and alanine require rigorous calibration to account for potential dose-rate dependencies and energy response variations [16,18]. Furthermore, macrodosimetric measurements often overlook microscopic heterogeneity in energy deposition, which are critical for understanding the differential responses of tumor and normal tissues. The unique pulse structures and instantaneous dose rates of FLASH beams can alter ionization density and track structure, influencing radical production and oxygen consumption dynamics—factors directly related to the FLASH effect [19,20].

Recent advances in detector technology have begun to address these challenges. Synthetic diamond detectors (e.g., PTW flashDiamond), with their high spatial resolution and minimal recombination effects, are promising for microdosimetric applications in FLASH electron and photon beams [21,22]. Similarly, silicon-based devices and single-event counting pixel detectors (e.g., Medipix) offer real-time, high-resolution dose mapping, enabling the characterization of beamlet interactions at submillimeter scales [23]. These tools, combined with Monte Carlo (MC) simulations, provide insight into the microenvironment of energy deposition, bridging the gap between physical dose delivery and biological efficacy [24,25]. Developing robust microdosimetric frameworks is essential to optimize FLASH-RT. By correlating microscopic dose distributions with radiobiological endpoints, such as DNA damage repair kinetics and oxygen enhancement ratios, microdosimetry can refine dose prescription paradigms and validate mechanistic hypotheses [13,26]. As FLASH RT transitions toward clinical implementation, integrating microdosimetric data into treatment planning systems will be crucial for maximizing therapeutic ratios and ensuring the safe, precise delivery of UHDR radiation [27,28].

### 2.2. Dose-Rate, Pulse Structure in FLASH-RT

**Figure 2:**
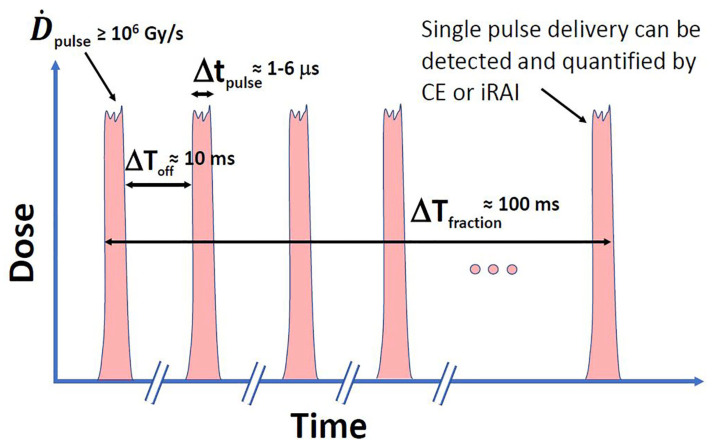
A schematic representation of the pulse structure in an idealized FLASH-RT beam, characterized by high instantaneous dose rates per pulse (${\dot{D}}_{\mathrm{pulse}}$) and ultrashort pulse durations ($\Delta t_{\mathrm{pulse}}$).

Beyond average dose rate, FLASH’s pulse structure—dose per pulse (DPP), pulse repetition frequency (PRF), and total beam-on time—critically shapes biophysical effects as illustrated in Figure 2 redrawn from El Naqa et al. [29]. This figure demonstrates that methods on optical imaging (e.g., Cerenkov emission (CE)) or ultrasound imaging (e.g., ionizing radiation acoustic imaging (iRAI)) can make single-pulse dosimetry feasible. For instance, ten pulses are required to deliver a 10 Gy fraction each 1–6 μs long and separated by roughly 10 ms (100 Hz repetition rate) with a per-pulse dose rate $\geq \;\;10^{6}$ Gy/s. This pulse microstructure provides multiple, short-duration checkpoints to detect any misalignment or errors between planned and delivered pulses and, if needed, halt the delivery process, which can be critical to avoid radiation-induced injuries [29,30,31]. Optical methods are more suited for superficial treatments (e.g., electron-based FLASH RT) while ultrasound methods can make measurements at deeper tissues (e.g., proton-based FLASH RT).

Preclinical investigations by Böhlen et al. [32] established that normal-tissue sparing exhibits a dose-dependent sudden effect transition (SET) in response to single-fraction irradiation, characterized by a threshold dose and an asymptotic sparing factor. Beyond this threshold, the FLASH-modifying factor (FMF) diminishes toward its minimum value, conferring pronounced normal-tissue protection under UHDR conditions. Complementing these findings, Grilj et al. [30] systematically evaluated pulsed electron FLASH-RT in murine models, identifying average dose rates (${\mathrm{DR}}_{av}$) as the dominant temporal beam parameter for preserving intestinal integrity. In their study, Grilj et al. irradiated C57BL/6 mice with a 17 Gy single-fraction dose using a prototype electron linear accelerator (LINAC), modulating PRF and DPP to decouple ${\mathrm{DR}}_{av}$ and DPP effects. Toxicity endpoints included overall survival and jejunal crypt regeneration at 96 h post-irradiation. Reducing ${\mathrm{DR}}_{av}$ while maintaining high DPP (>1 Gy/pulse) exacerbated intestinal damage and mortality, whereas elevating ${\mathrm{DR}}_{av}$ to ≥100 Gy/s—even at constant DPP—maximized FLASH sparing. This threshold ${\mathrm{DR}}_{av}$ of 100 Gy/s emerged as critical for mitigating radiation-induced crypt loss and improving survival, independent of DPP variations. These results align with Böhlen et al.’s framework [32], wherein surpassing a dose-rate or DPP threshold triggers nonlinear biological sparing. Grilj et al. further demonstrate that ${\mathrm{DR}}_{av}$, rather than instantaneous dose rate or DPP alone, governs the FLASH effect in pulsed beams. Their findings underscore ${\mathrm{DR}}_{av}$ as a scalable parameter for clinical FLASH-RT systems, providing a pragmatic benchmark for accelerator design. Together, these studies delineate two interlinked prerequisites for FLASH efficacy: (1) a threshold dose or dose-rate to activate the SET mechanism and (2) sustained ${\mathrm{DR}}_{av}$ ≥ 100 Gy/s to maintain tissue protection, irrespective of the beam pulsing structure.

## 3. Free Radicals and Effect-Modifying Molecules

FLASH-RT generates an intense burst of free radicals ${(}^{•}\mathrm{OH}$, $e_{\mathrm{aq}}^{-}$, $H^{•}$, $O_{2}^{•-}$) over micro- to millisecond timescales. These radicals drive both direct biomolecular damage and secondary chain reactions (e.g., lipid peroxidation), but the presence of effect-modifying molecules (endogenous antioxidants, small reactive species, and exogenous radioprotectors) can dramatically alter net biological outcomes.

### 3.1. Radiolysis of Water and Primary Reactive Species

The radiolysis of water mediated by FLASH-RT constitutes a central mechanism underlying the distinct biological effects associated with UHDR radiation. Ionizing tracks deposit energy that ionizes and excites water, yielding primary reactive species. The main products are hydrated electrons ($e_{\mathrm{aq}}^{-}$), hydroxyl radicals ${(}^{•}\mathrm{OH}$), and hydrogen atoms ($H^{•}$), along with molecular products like hydrogen gas ($H_{2}$) and hydrogen peroxide ($H_{2}O_{2}$) [33]. For example, one generalized radiolysis scheme is:

(1) $$H_{2}O\overset{\gamma }{\to }e_{\mathrm{aq}}^{-}+H^{•}{+\;}^{•}\mathrm{OH}+\left(H_{2},\;H_{2}O_{2},\;{\mathrm{HO}}_{2}^{•},\;\ldots \right)$$

where radical yields depend on LET and dose rate. These radicals initiate a cascade: $e_{\mathrm{aq}}^{-}$ and $H^{•}$ consume $O_{2}$ to form superoxide and OH•, while OH• and $H^{•}$ attack biomolecules or recombine. In oxygenated cells, OH• reacting with DNA is typically irreversibly fixed by O_2_ to yield stable damage [34]. Thus, in CONV-RT, indirect DNA damage by OH• is enhanced by oxygen. In FLASH-RT, the altered radical kinetics (very high instantaneous OH•) lead to higher recombination (e.g., 2 OH•→H2O2, or OH•+eaq−→HO−), reducing net effective OH• concentrations [34,35].

### 3.2. Temporal and Spatial Kinetics of Radical Chemistry

Primary radicals in aqueous solution have lifetimes on the order of microseconds (a few μs in bulk water) and diffuse only nanometers before reacting [33]. At conventional dose rates (∼0.1Gy/s), ionization events are spatially isolated, so radicals (OH•,eaq−) diffuse to biomolecules and induce damage [36]. Under FLASH-RT, ionization events overlap extensively, driving radicals to recombine (e.g., OH•+•OH→H2O2; ${\mathrm{ROO}}^{•}+{\mathrm{ROO}}^{•}\to$ non-radical products) before reaching targets [35,37]. A computational physicochemical model confirms that radical–radical recombination rates at UHDR are up to an order of magnitude higher than at conventional rates, reducing net ROS yields [35,38].

### 3.3. Radiolytic Oxygen Depletion (ROD) and Transient Hypoxia

**Figure 3:**
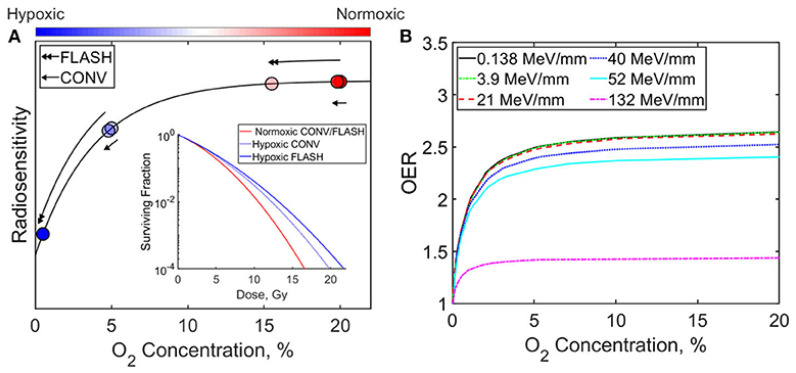
Graphs illustrate the impact of oxygen concentration and radiation delivery technique (FLASH vs. CONV) on (**A**) radiosensitivity and (**B**) the oxygen enhancement ratio (OER) [43].

FLASH pulses consume dissolved molecular O_2_ faster than blood perfusion that can reoxygenate tissue via rapid reactions with radiolytic radicals ($e_{\mathrm{aq}}^{-}$ + $O_{2}\to O_{2}^{•-};\;H^{•}+O_{2}\to {\mathrm{HO}}_{2}^{•}$), transiently lowering ${\mathrm{pO}}_{2}$ [39,40]. Moreover, Monte Carlo simulations show that radical recombination conserves much of the oxygen, limiting net depletion [41]. Thus, ROD likely contributes partially in normal tissue sparing but cannot be the dominant mechanism [42]. Figure 3, redrawn from Ashraf et al. (2020) [43], illustrates the influence of oxygen concentration and radiation delivery method (FLASH vs. CONV) on radiosensitivity and the oxygen enhancement ratio (OER). As shown, increasing oxygen concentration generally enhances both radiosensitivity and OER. Furthermore, the relative efficacy of FLASH and CONV techniques varies depending on whether the cellular environment is hypoxic or normoxic.

### 3.4. Interaction with Effect-Modifying Molecules

Endogenous antioxidants—including glutathione (GSH), ascorbate (${\mathrm{AH}}^{-}$), and tocopherols (TOH)—alongside rapid production of small reactive species (NO•), biomolecules radicals (R•), and peroxyl radicals (${\mathrm{ROO}}^{•}$) intercept, causing neutralizing interactions in which they are critical in determining the efficacy and safety of FLASH-RT outcomes [35]. Monte Carlo modeling (IONLYS-IRT) incorporating GSH, ${\mathrm{AH}}^{-}$, NO•, and TOH shows that antioxidants compete effectively rather than radical–radical recombination, quenching peroxyl radicals and preventing lipid peroxidation [35].

## 4. In Silico and Sensor Tools for Tumor/Normal Microenvironment

In silico and sensor-based tools are crucial for understanding the microenvironmental dynamics driving the FLASH effect, these tools bridge molecular radiochemistry and whole-tissue physiology, guiding the optimization of FLASH-RT protocols.

### 4.1. In Silico Modeling of FLASH Microenvironment

#### 4.1.1. Monte Carlo Track-Structure Simulations

Monte Carlo track-structure codes such as Geant4-DNA simulate individual ionization events and subsequent water radiolysis and inter-track chemistry at nanometer scales, predicting yields of OH•, $e_{\mathrm{aq}}^{-}$, and $H^{•}$ radicals under FLASH dose rates [15,44]. Extensions to TOPAS-nBio incorporate inter-track interactions, showing up to an order-of-magnitude increase in radical–radical recombination rates at UHDR, thereby reducing net ROS yields [45,46]. These simulations also model DNA damage clustering differences between conventional and FLASH pulses, providing mechanistic insight into differential normal-tissue sparing.

In summary, track-structure simulations offer detailed predictions of radical yields and spatial clustering that underpin the FLASH effect. However, they remain limited by the absence of direct experimental validation in complex biological media and by simplified assumptions regarding their cellular geometry and diffusion. Future work should couple such simulations with live-cell measurements of ROS dynamics and include explicit modeling of chromatin organization and subcellular compartments to bridge the gap between physical predictions and observed tissue responses.

#### 4.1.2. Reaction–Diffusion PDE Models

Partial differential equation (PDE) models, particularly reaction–diffusion systems, are used to simulate the spatiotemporal dynamics of oxygen concentration in biological tissues. In oncology, these models integrate key factors like vascular geometry, metabolic consumption, and ROD to predict transient hypoxia in tumors and rapid reoxygenation in normal tissues during FLASH-RT [35,47,48]. Continuum reaction–diffusion frameworks of tumor hypoxia and reoxygenation couple tissue oxygen transport with radiolytic consumption and metabolic uptake. The Transport of Oxygen Dynamics (TOD) model quantifies O_2_ reaction kinetics with radicals and rediffusion from vasculature, predicting only modest net ROD at clinically relevant doses [47,49]. Phenomenological 1D–3D models integrate vascular geometries and dose-rate parameters to simulate oxygen enhancement ratio (OER) modulation during FLASH, concluding that ROD alone cannot fully explain the FLASH effect [48].

Overall, PDE-based approaches capture tissue-scale oxygen dynamics and suggest that transient radiolytic oxygen depletion contributes only partially to the FLASH effect. Yet, they often neglect dynamic perfusion changes, heterogeneity of vascular networks, and interactions with other radical species. To strengthen mechanistic understanding, experimental measurements of pO_2_ transients in vivo (e.g., via fiber-optic oximetry) should be used to validate model predictions, and future models must integrate blood flow dynamics and the heterogeneous tissue microarchitecture.

### 4.2. Sensor Technologies for Real-Time Microenvironment Mapping

#### 4.2.1. Optical Oxygen Probes and Fiber-Optic Oximetry

Phosphorescent Oxyphor molecule, Pd- or Pt-porphyrin-based nanoprobes enable oxygen imaging via quenching of triplet lifetimes, achieving temporal resolutions up to 3.3 kHz during proton FLASH [50]. Water-soluble phosphorescent nanoparticles combined with fiber-optic instruments have measured in vitro O_2_ kinetics at 200 Hz under UHDR, validating computational ROD predictions [51]. These systems allow sub-millisecond tracking of pO_2_ in cell suspensions and tissue phantoms.

These optical probes provide exceptional temporal resolution for monitoring rapid oxygen changes during FLASH pulses. However, most studies are limited to simplified phantom or cell-suspension models under ambient oxygen levels. There is a need for in vivo validation in tissues at physiological pO_2_ levels, alongside calibration against complementary methods (e.g., photoacoustics), to ensure quantitative accuracy and to determine how oxygen dynamics correlate with biological endpoints.

#### 4.2.2. Photoacoustic Imaging

Photoacoustic lifetime (PALT) imaging employs oxygen-sensitive dyes to map hemoglobin saturation and dissolved O_2_ in tumors with 200 μm spatial resolution [52]. In vivo demonstrations have shown dynamic changes in oxygenation post-FLASH in murine tumor models, correlating with treatment efficacy [53]. Photoacoustic modalities thus can offer label-free, high-resolution mapping of vascular responses to UHDR.

Photoacoustic methods uniquely combine depth penetration with oxygen sensitivity, revealing localized vascular responses to FLASH. Nonetheless, limitations include potential signal confounds from hemoglobin concentration and laser fluence variability. Future work should establish standardized quantification protocols, integrate multi-wavelength imaging for absolute pO_2_ levels, and validate findings against invasive oxygen electrodes.

#### 4.2.3. Ionizing-Radiation Acoustic Imaging (iRAI)

iRAI leverages acoustic waves generated by rapid thermoelastic expansion upon radiation absorption to reconstruct 3D dose distributions in real time. Studies show linear correlation between iRAI signal amplitude and delivered dose, enabling deep-tissue dosimetry during single FLASH pulses [54]. Volumetric imaging systems using matrix array transducers have achieved frame rates sufficient for UHDR verification in clinical settings [55]. Ba Sunbul et al. used Monte Carlo-based simulations and the Matlab k-Wave toolbox to model the FLASH-RT using iRAI [56].

iRAI offers a promising window into dose deposition in deep tissues without exogenous contrast. However, its spatial resolution and sensitivity under heterogeneous tissue properties require further characterization. Experimental benchmarking against gold-standard dosimeters and exploration of combined iRAI–optical approaches will be essential to confirm its accuracy for clinical FLASH QA purposes.

#### 4.2.4. Microfluidic and Organ-on-Chip Platforms

Microphysiological systems and organ-on-chip platforms integrate living human cells within microfluidic channels to recapitulate key aspects of tissue architecture, mechanical forces, and biochemical gradients. Incorporated electrochemical and optical sensors enable controlled FLASH exposures in physiologically relevant 3D environments [57]. Such devices provide a controlled microenvironment in which pH, dissolved oxygen, redox potential, and other critical parameters can be monitored in real time during irradiation, making them powerful tools for dissecting the FLASH effect [58]. The human Lung Alveolus-on-Chip—originally developed by Huh et al. [59]—consists of two parallel microchannels separated by a porous, flexible membrane coated with primary human alveolar epithelial cells on one side and pulmonary microvascular endothelial cells on the other, all subject to cyclic strain to mimic breathing motions [59]. Dasgupta et al. recently adapted this platform to study acute radiation-induced lung injury under UHDR exposures, measuring epithelial barrier integrity via trans-epithelial electrical resistance (TEER) sensors and quantifying cytokine release in the perfusate post-FLASH [60]. Radiotherapy-on-Chip platforms embed patient-derived colorectal cancer organoids in a 3D matrix within microfluidic channels outfitted with impedance sensors (for cell viability and barrier integrity) and optical pH/O_2_ probes, enabling real-time monitoring during FLASH irradiation [61]. By combining these computational and experimental approaches, researchers can iteratively validate their models, refine mechanistic hypotheses (e.g., about ROD vs. radical recombination), and optimize FLASH-RT parameters for both tumor control and normal-tissue protection.

Organ-on-chip systems can bridge the gap between in vitro simplicity and in vivo complexity, offering physiologically relevant microenvironments under UHDR. Yet, challenges include limited throughput, integration of immune components, and translation of readouts to whole-organ responses. Future development should focus on higher-density platforms, incorporation of vascular perfusion, and correlation with animal-model outcomes to validate these microphysiological insights.

## 5. In Vitro vs. Ex Vivo vs. In Vivo Measurements

Characterizing the tumor microenvironment (TME) under FLASH irradiation requires complementary models—from simplified cell cultures to intact organisms—to capture biochemical, physiological, and systemic responses. In vitro assays allow precise control over oxygen tension, radical scavengers, and molecular endpoints (e.g., ROS generation, DNA damage), while ex vivo tissue preparations preserve native architecture and perfusion elements for short-term functional studies. In vivo models integrate full vascular, immune, and metabolic networks to assess clinically relevant endpoints (e.g., tumor growth delay, immune cell infiltration). Collectively, these measurements may describe the mechanisms of the FLASH effect—specifically, the tissue-sparing properties under UHDR irradiation—through precise characterization of its biophysical and molecular dynamics. Such insights could directly inform the optimization of translational FLASH-RT protocols for future clinical trials.

### 5.1. In Vitro Models

In vitro systems offer precise control over environmental parameters—such as oxygen tension and radical scavenger concentrations—and enable high-throughput mechanistic studies of FLASH-RT effects on tumor cells. Two-dimensional monolayer cultures and 3D spheroids have both been employed to dissect the radiobiology of FLASH irradiation.

#### 5.1.1. Oxygen Dynamics and Radiolytic Yields

Recent in vitro studies have established that the FLASH effect is intrinsically oxygen-dependent, with its radioprotective outcomes critically influenced by oxygen tension [62,63,64,65]. These investigations into oxygen dynamics during FLASH irradiation challenge the hypothesis that ROD alone accounts for normal-tissue sparing. In vitro studies using cultured cells under controlled O_2_ conditions demonstrate that transient hypoxia induced by FLASH pulses is minimal and short-lived [66]. Advanced techniques such as fluorescence/phosphorescence lifetime imaging microscopy (FLIM/PLIM) that offer subcellular resolution for mapping oxygen gradients [67,68] and electron paramagnetic resonance (EPR) oximetry reveal that UHDR irradiation causes sub-second O_2_ reductions of only dips of a few mmHg in cell suspensions, with full recovery within seconds post-pulse [64,69].

These in vitro measurements confirm that FLASH pulses induce only transient, small-magnitude oxygen depletion in cell suspensions, suggesting ROD is not the sole driver of the FLASH effect. However, they lack validation under physiologically relevant pO_2_ conditions and do not account for tissue architecture or perfusion. Future experiments should extend these measurements to 3D cultures and ex vivo tissues at physiological oxygen tensions, and correlate real-time oximetry with downstream biological endpoints.

#### 5.1.2. ROS Generation and DNA Damage

The mechanistic basis of FLASH-mediated tissue sparing is hypothesized to stem from UHDR-induced radical–radical recombination, where the transient surge of free radicals leads to mutual annihilation, thereby reducing indirect DNA damage [47,47]. To probe these fleeting radicals, in vitro models employ fluorogenic ROS sensors (e.g., DCFDA) and EPR coupled with spin traps, which stabilize short-lived species for detection [70,71]. Immuno-spin trapping further enhances sensitivity, enabling identification of DNA-bound radicals [72]. Post-irradiation oxidative stress, a stable proxy for radical damage, is assessed via DNA strand breaks (Comet assay), γH2AX foci for double-strand breaks, and lipid peroxidation assays using fluorogenic lipophilic probes [73,74]. These methodologies collectively elucidate how FLASH irradiation minimizes ROS-mediated genomic injury in normal tissues while maintaining cytotoxic efficacy in tumors, underscoring the pivotal role of controlled oxygen environments and radical chemistry in optimizing FLASH-RT protocols.

These assays demonstrate reduced ROS-mediated DNA damage under FLASH versus CONV-RT dose rates, supporting radical recombination models. Nevertheless, most studies are conducted in oversimplified monolayer cultures and lack direct comparison with in vivo oxidative markers. Incorporating 3D tissue constructs and coupling ROS measurements with functional readouts (e.g., cell viability, senescence) will be critical to validate these mechanisms under more realistic conditions.

#### 5.1.3. Clonogenic Survival Assays

The clonogenic assay remains the gold standard for quantifying reproductive cell death after irradiation [75]. It captures all forms of radiation-induced lethality (mitotic catastrophe, apoptosis, necrosis, etc.) by measuring a cell’s ability to form colonies, serving as an in vitro surrogate for tumor sterilization in vivo [76]. Early studies described “hockey-stick” survival curves under normoxia, where FLASH and CONV dose rates diverged at higher doses (>7–10 Gy), suggesting FLASH sparing [77,78]. However, reproducibility was inconsistent [79,80], prompting investigations into oxygen dependency. Hypoxia lowered the dose required for survival curve “breaks,” linking FLASH effects to oxygen depletion [79,80,81,82]. Later studies found no differences in normoxia or anoxia [83,84], while recent work reports mixed outcomes: FLASH spared H454 glioblastoma [62] and some human lines [85] but reduced survival in murine pancreatic cancer cells [86] or showed no effect in A549 and IMR90 cells [63,87]. Hypoxia consistently enhanced FLASH sparing in DU145 and A549 spheroids [63,88]. Despite challenges, the clonogenic assay remains indispensable for FLASH research. Collaborative studies across mechanistic investigations (e.g., oxygen scavengers) are critical to unravel FLASH biology.

Clonogenic assays confirm that FLASH-RT can spare clonogenic potential in some cell types, but inconsistencies arise from divergent oxygen conditions and assay protocols. Standardized oxygen control, dose regimens, and cross-laboratory comparisons are needed to establish robust survival benchmarks and clarify the cell-specific determinants of the FLASH-RT sensitivity.

### 5.2. Ex Vivo Models

Ex vivo tissue models preserve native extracellular matrix architecture, partial vasculature, and cell–cell interactions, allowing short-term functional assessment of FLASH-RT in a quasi-physiological context.

#### 5.2.1. Organotypic Slice Cultures (OSC)

Thin tumor or normal tissue slices (200–400 μm) maintain multicellular complexity. Mouse lung slices exposed to FLASH pulses reveal a significantly higher proportion of replicating cells after FLASH versus CONV irradiation, robustly demonstrating the normal-tissue-sparing FLASH effect in OSCs and facilitating rapid, medium-throughput toxicity screening without requiring additional animal use. Studies show dose-dependent reductions in cell division and viability—measured via histological markers and live-dead staining—while cytokine release (e.g., IL-6, TNF-α) in perfusate can be quantified, facilitating rapid toxicity screening without full animal use [89].

OSC models bridge in vitro and in vivo contexts by preserving the tissue architecture, yet they are limited by short viability windows and lack of immune and systemic factors. Extending culture longevity, incorporating perfusion bioreactors, and validating OSC responses against in vivo endpoints will enhance their translational relevance.

#### 5.2.2. Microelectrode and Optical Measurements

Ex-vivo studies combining microelectrode recordings and optical measurements have established a multi-modal framework for probing the biophysical mechanisms of the FLASH effect: in acute rodent hippocampal slices, stable field excitatory postsynaptic potential (fEPSP) amplitudes, slopes, and paired-pulse facilitation ratios recorded via 32-channel flexible perforated microelectrode arrays during UHDR FLASH-RT, which contrast sharply with the attenuation seen under CONV-RT dose rates [90]. Another mechanism utilizes optical measurements: concurrent Cherenkov emission imaging during proton and electron FLASH pulses, which provides spatially resolved maps of dose deposition and free-radical generation in ex vivo samples [91,92] and diffuse optical spectroscopy of ex vivo skin and muscle further underscores the importance of accurately characterizing tissue absorption and scattering coefficients to interpret luminescence-based measurements [93,94]. These ex vivo findings demonstrate the preservation of long-term potentiation and cognitive outcomes following FLASH-RT.

These multi-modal techniques offer high-resolution biophysical insights in intact tissue, but their translation to clinically relevant organs and time scales remains unexplored. Integrating long-term functional assays and correlating electrical/optical readouts with histopathological outcomes will be important next steps.

### 5.3. In Vivo Models

Recent in vivo investigations consistently demonstrate that UHDR FLASH-RT markedly spares normal tissues while preserves tumor control efficacy, a phenomenon termed the “FLASH effect” [3]. In murine skin models, FLASH-RT reduces fibrosis, epidermal contraction, and collagen deposition compared to conventional dose-rate RT CONV-RT [95]. In vivo gastrointestinal studies reveal significantly lower lipid peroxide accumulation in intestinal tissues post-FLASH and correlate with reduced mucosal injury [96,97]. Neurocognitive assessments further show long-term preservation of learning and memory in rodents receiving cranial FLASH-RT versus CONV-RT [62]. Mechanistic in vivo work corroborate that transient radiochemical oxygen depletion, attenuated ROS bursts, and maintenance of mitochondrial integrity as central drivers of normal-tissue sparing [98,99]

In vivo models provide compelling evidence of the FLASH effect across multiple organs and species, yet mechanistic biomarkers (e.g., real-time ROS, oxygen transients) are rarely measured in situ. Incorporating intravital imaging and molecular biosensors, alongside standardized functional endpoints, will be essential to link biophysical processes to clinical outcomes.

## 6. Example Use Cases (Pre-Clinical and Clinical)

### 6.1. Pre-Clinical Trials

#### 6.1.1. Brain/CNS

Mouse studies of cranial irradiation provide strong evidence of lower oxidative injury with FLASH. Montay Gruel et al. [62] delivered 10 Gy to mouse brains using X-rays (FLASH: ∼100 Gy/s vs. CONV: 0.07 Gy/s) and found that FLASH spared neurocognitive function while CONV caused lasting deficits [62]. Correspondingly, FLASH-treated brains showed significantly lower H_2_O_2_ levels and almost no neuroinflammation (microglial activation) relative to CONV [62]. In these mice, FLASH also preserved neuronal morphology and synaptic density, whereas CONV induced dendritic loss and astrogliosis (an oxidative-stress marker) [62]. Complementing this, Limoli and colleagues reported that FLASH whole brain irradiation elicited far less oxidative DNA damage and blood–brain barrier disruption than CONV, preserving synaptic integrity [100]. In juvenile mice, hypofractionated FLASH similarly attenuated gliosis and vascular injury. Together, these studies imply that FLASH produces less diffusible ROS or more rapidly quenches them in brain tissue, thereby reducing downstream peroxidation and inflammatory signaling [100].

#### 6.1.2. Lung

Thoracic FLASH markedly reduces radiation induced pulmonary injury. Fouillade et al. [101] found that a single 17 Gy FLASH electron dose (∼100 Gy/s) to mouse lungs (vs. CONV 0.1 Gy/s) preserved lung progenitor cells and halved the incidence of late senescence [101]. Transcriptomic analyses showed that FLASH minimized upregulation of pro inflammatory genes (e.g., EGR1, TGF-β1, NF-κB) after irradiation [101]. In consequence, FLASH irradiated lungs had much fewer senescent cells and DNA-damaged cells at late times than CONV, suggesting more complete repair. These findings were supported by histology: FLASH lungs showed minimal inflammation and fibrosis, whereas CONV lungs developed thickened septa and collagen deposition [101].

#### 6.1.3. Intestine/Abdominal

UHDR abdominal irradiation likewise attenuates oxidative injury. Zhu et al. [102] irradiated mice (BALB/c nude) with whole abdomen 6 MV X-rays (FLASH: >150 Gy/s vs. CONV: 0.1 Gy/s, single 10–15 Gy). FLASH-treated mice exhibited markedly less acute intestinal mucosal damage and faster recovery than with CONV [102]. Blood tests showed fewer inflammatory leukocytes and lower TNF-α/IL-6 chronically in FLASH animals. Strikingly, ROS probe signals in intestine were higher immediately after FLASH than CONV, yet lipid peroxidation (malondialdehyde) was significantly lower with FLASH [102]. This suggests that FLASH-triggered ROS are quickly neutralized (by antioxidants) before initiating lipid damage. Overall, FLASH dramatically reduced markers of oxidative stress and inflammation in gut tissue. Earlier work similarly showed that abdominal FLASH (electron or X-ray) preserves intestinal crypts and reduces serum inflammatory markers (TNF-α, IL-6) relative to CONV RT [6].

#### 6.1.4. Skin and Other Tissues

FLASH also mitigates radiation damage in skin and other tissues, though oxidative endpoints have been less studied. In murine skin, Soto et al. [103] found much lower incidence of moist desquamation and collagen fibrosis after electron FLASH (180 Gy/s) than CONV. Allen et al. [100] similarly reported no acute dermatitis and reduced late depilation with whole body FLASH compared to CONV [104]. These phenotypic sparing effects imply lower local oxidative stress in skin, consistent with observations of reduced inflammatory infiltrates. In breast, heart, and other models, FLASH likewise prevents capillary loss and inflammatory cytokine expression seen with CONV. By contrast, preclinical tumor studies show comparable or enhanced ROS damage with FLASH; for example, multiple cell-line xenografts exhibit similar DNA damage and killing with FLASH vs. CONV (consistent with maintained tumor control). The tissue selectivity of FLASH (protecting normal but not tumor) is often attributed to tumor cells’ higher baseline oxidant load and iron-driven Fenton chemistry—making them less able to capitalize on the brief radical recombination in FLASH [8,105].

### 6.2. Clinical Trials

#### 6.2.1. First-in-Human Electron FLASH RT

The first in-human application of FLASH RT was reported by Bourhis et al. [7] (2019), who administered a single 15 Gy in 90 ms fraction of 5.6 MeV electron-based FLASH RT to a 75-year-old patient with cutaneous T-cell lymphoma, demonstrating procedural feasibility and absence of acute toxicity.

#### 6.2.2. FAST-01: Proton FLASH for Extremity Bone Metastases

FAST-01 is a prospective, single-center, nonrandomized phase I study enrolling 10 adult patients (age range 27–81 years) with 1–3 painful bone metastases in the extremities (excluding hands, feet, and wrists) and life expectancy >2 months, without fractures or prior radiotherapy at the treatment site(s). Each bone metastases treated with a single 8 Gy fraction of proton FLASH RT delivered at ≥40 Gy/s using a FLASH-enabled Varian ProBeam system [106]. Protocol assessments included workflow feasibility, pain response, and acute treatment-related toxicities up to 3 months post-treatment, with no dose-limiting toxicities observed. Clinical outcomes revealed pain relief in 67% of treated sites at 1 month and complete response in 50% of sites, with no unexpected adverse events reported [106].

#### 6.2.3. FAST-02: Proton FLASH for Thoracic Bone Metastases

Following FDA IND approval being granted, the FAST-02 trial was initiated to treat symptomatic metastatic lesions of the thoracic bones in 10 adult patients with 1–3 painful non-spinal thoracic bone metastases, utilizing the same 8 Gy single-fraction proton FLASH RT regimen at UHDR [107]. Follow-up assessments occur on treatment day, day 7, day 15, months 1–3, and every 6 months thereafter. The study is anticipated to be completed by 1 May 2027, reflecting a 2.5–4 year timeline encompassing enrollment through data analysis. This study aims to assess safety, pain palliation, and workflow parameters in a more anatomically challenging region, leveraging transmission-mode proton beams to achieve ultrahigh dose rates in a clinical setting [107].

The long-term safety profile of FLASH-RT remains uncertain, as pivotal trials like FAST-02 (completion 2027) are currently limited to 2.5–4-years follow-ups, leaving late effects (≥5 years) entirely unexplored. Extended follow-up in such studies will be essential to determine whether FLASH’s protective mechanisms translate into sustained safety in humans or unveil latent risks not captured in the current short-term assessments.

## 7. Current Challenges

FLASH-RT delivers therapeutic doses at UHDR; (≥40 Gy/s) to exploit a normal-tissue–sparing “FLASH effect” while maintaining tumor control. However, its clinical translation is impeded by (1) technical challenges in dosimetry accuracy and beam-parameter reproducibility [95,108] (2) treatment-planning system (TPS) and quality-assurance (QA) gaps [108,109], (3) incomplete mechanistic understanding of the FLASH effect (oxygen depletion, radical chemistry) [9,108], (4) preclinical–clinical translation issues (model variability, tissue hypoxia) [110], and (5) economic, regulatory, and infrastructural barriers (equipment cost, training, approval pathways) [111]. A consolidated overview of the major challenges and corresponding gaps in FLASH-RT biophysics and clinical translation is summarized in Table 1 and is discussed below.

### 7.1. Technical Dosimetry and Beam Delivery

#### 7.1.1. Dosimetric Accuracy of UHDR

Accurate dose and dose-rate measurements at FLASH-RT level intensities remains challenging: conventional ion chambers and diodes suffer from dose-rate-dependent response and saturation effects under UHDR conditions [109]. Newer dosimeters, primarily parallel plate are designed to address these issues [112].

#### 7.1.2. Beam Parameter Characterization and Reproducibility

The magnitude of the FLASH-RT effect is highly sensitive to beam pulse structure, mean dose-per-pulse, and total dose rate; yet many UHDR systems lack real-time monitoring of these parameters, leading to inter-institutional variability and compromised reproducibility [113,114].

### 7.2. Treatment Planning and Quality Assurance

#### 7.2.1. Treatment Planning System Adaptation

Existing treatment planning system (TPS) algorithms are calibrated for conventional dose rates (0.5–5 Gy/min) and do not model UHDR-specific interactions (e.g., rapid oxygen depletion). Without dedicated UHDR modules, dose calculations may be inaccurate for FLASH-RT fields [108]. This would require the development of dedicated planning systems that can take dose rate optimization as part of their design.

#### 7.2.2. QA Frameworks

There is currently no consensus on quality assurance (QA) protocols for UHDR delivery. Conventional QA phantoms and workflows must be adapted or redesigned to verify beam flatness, symmetry, and output constancy at ≥40 Gy/s [109,115], which constitute higher risk mitigation requirements.

### 7.3. Biological Mechanisms and Preclinical Models

#### 7.3.1. Oxygen Depletion Hypothesis

One leading hypothesis posits that FLASH-RT rapidly depletes molecular oxygen, transiently creating hypoxia that protects normal tissues. However, in vitro experiments at high oxygen tensions (ambient $O_{2}$ concentration 21%) often fail to replicate the effect, indicating a need to study oxygen kinetics under physiologically relevant tensions (4–7%) [9,116].

#### 7.3.2. Radical Chemistry and Alternative Mechanisms

Physicochemical models suggest peroxyl radical recombination and antioxidant pathways may also play critical roles in the FLASH effect. Yet, these mechanisms remain under-investigated in vivo, demanding comprehensive molecular and imaging studies [37].

#### 7.3.3. Preclinical Model Variability

Preclinical demonstrations of normal-tissue sparing span multiple species and endpoints but use heterogeneous beam modalities (electrons, protons, heavy ions) and dose regimens. This variability complicates direct translation; standardized in vivo protocols are urgently required [117].

### 7.4. Clinical Translation Challenges

#### 7.4.1. Patient Selection and Clinical Endpoints

Current human trials (e.g., FAST-01/02) [106,107] focus on palliative bone metastases with single-fraction endpoints (pain relief). Expansion to curative indications and incorporation of long-term functional outcomes will necessitate careful patient stratification and endpoint harmonization.

#### 7.4.2. Equipment and Infrastructure

Most clinical linear accelerators (LINACs) cannot deliver UHDR; retrofitting existing machines is complex, and very high energy electron (VHEE) or proton therapy platforms are costly and are limited in availability [118]. Institutions must assess trade-offs between electron, proton, and emerging VHEE sources for deep-seated tumors.

### 7.5. Regulatory, Economic, and Logistical Barriers

#### 7.5.1. Cost and Training

Specialized UHDR hardware, dosimetry devices, and facility upgrades impose substantial capital expenditure. Additionally, physicists and therapists require new training programs for UHDR operation and safety procedures [109].

#### 7.5.2. Regulatory Pathways

FLASH-RT currently lies outside established radiotherapy regulatory frameworks. Early engagement with regulatory authorities, such as the U.S. Food and Drug Administration (FDA) and the European Medicines Agency (EMA), will be essential to define the necessary requirements for investigational device exemption (IDE) applications or CE marking. Additionally, coordinated efforts among stakeholders to draft consensus guidelines will be critical to facilitate clinical translation [110].

**Table 1:** Summary of Major Challenges and Gaps in FLASH-RT and Translation.

Challenge	Key Issue	Gap/Need	Source
Dosimetry and Detection	Detector saturation and dose-rate dependency at UHDR levels	Development of high-speed, FLASH-compatible dosimeters and redesigned phantoms	[109,112]
Beam Reproducibility	Inconsistent pulse structure and lack of real-time beam monitoring	Standardization of beam delivery parameters and inter-center reproducibility protocols	[113,114]
Treatment Planning Systems (TPS)	Conventional TPSs do not model FLASH-specific physics or oxygen dynamics	Dose-rate–aware TPS algorithms with oxygen-depletion models	[108]
Quality Assurance (QA)	No validated QA tools or protocols at FLASH dose rates	QA frameworks and phantoms specific to ≥40 Gy/s with real-time verification tools	[109,115]
Biological Mechanisms	Incomplete modeling of ROS, oxygen depletion, and radical interactions	Multi-scale in vitro/in vivo/in silico integration for mechanistic discovery	[9,37]
Preclinical Models	Heterogeneity in species, beam types, and outcome endpoints	Standardized animal models and study protocols	[117]
Clinical Trial Design	Focused mainly on palliative care with limited long-term data	Trials addressing curative intent and long-term toxicity, functional endpoints	[106,107]
Infrastructure	Limited access to FLASH-capable platforms (LINACs, protons, VHEE)	Strategic investment aligned with tumor site requirements	[118]
Training and Operations	Operational unfamiliarity and lack of clinical FLASH experience	Education, credentialing, and FLASH-specific clinical training pathways	[109]
Regulation	Absence of specific regulatory frameworks for FLASH devices and protocols	Engagement with FDA/EMA and establishment of approval and safety standards	[110]

## 8. Recommendations

Based on the aforementioned challenges, we identify a set of strategic recommendations aimed at addressing key gaps in beam characterization, treatment planning, quality assurance, mechanistic research, and regulatory alignment. These recommendations are summarized in Table 2 and are intended to guide stakeholders across research institutions, clinical centers, industry, and regulatory bodies in building a sustainable and evidence-based foundation for FLASH-RT in the clinic.

### 8.1. Standardization of Beam Characterization Protocols

Establish consensus guidelines for the measurement of key beam parameters, including dose per pulse, pulse repetition frequency, and mean dose rate, across different delivery platforms. Harmonized protocols will ensure consistency, reproducibility, and comparability of FLASH-RT data generated at various institutions.

### 8.2. Implementation of Dedicated UHDR-TPS Modules

Incorporate models of oxygen depletion and radiation-induced radical chemistry into UHDR treatment planning algorithms to enable accurate prediction of FLASH effects. These dedicated TPS modules will support biologically informed dose calculations, facilitating optimized treatment designs that account for the unique radiobiological mechanisms underlying FLASH-RT.

### 8.3. Establishment of Robust QA Frameworks

Develop UHDR-compatible phantoms and high-resolution detectors tailored for FLASH-RT beam properties. Standardize QA procedures through collaborative efforts led by professional bodies such as the American Association of Physicists in Medicine (AAPM) and the European Society for Radiotherapy and Oncology (ESTRO) task groups, ensuring reliability, safety, and consistency across clinical and research settings.

### 8.4. Advancement of Mechanistic Research

Undertake multicenter, standardized preclinical studies conducted under clinically relevant oxygen tensions to elucidate the biological mechanisms underlying FLASH-RT. Integrate molecular, imaging, and functional endpoints to comprehensively assess tissue response, enabling the identification of biomarkers and refinement of therapeutic models for clinical translation.

### 8.5. Forge Preclinical Consortia

Coordination of interinstitutional networks to facilitate the sharing of beam time, standardized protocols, and preclinical data. Such collaborative consortia will enhance reproducibility, enable large-scale meta-analyses, and accelerate the validation of FLASH-RT mechanisms and outcomes across diverse experimental settings.

### 8.6. Early Engagement with Regulatory Authorities

Collaborate proactively with FDA and the EMA to establish clear UHDR device classifications, dosimetry standards, and clinical-trial design criteria. Simultaneously, develop grant-supported training initiatives to build regulatory expertise and ensure workforce readiness for FLASH-RT implementation.

### 8.7. Economic and Infrastructure Planning

Conduct comprehensive cost–benefit analyses of electron, proton, and VHEE delivery platforms, and advocate for public–private partnerships to mitigate upfront capital and operational expenditures.

## 9. Conclusions

FLASH-RT is a promising technology for better targeting the cancer while sparing the surrounding normal tissue. However, many biophysical factors that affects the microenvironment remain unknown. We summarize current challenges and make recommendations to address them, emphasizing the role of emerging computational and imaging technologies that can bridge this gap leading to better understanding of underlying radiobiology and safer clinical implementation.

## Figures and Tables

**Table 2:** Summary of Recommendations for Advancing FLASH-RT Implementation.

Recommendation Area	Proposed Action	Objective/Outcome
Standardization of Beam Characterization	Establish consensus protocols for measuring dose per pulse, repetition frequency, and mean dose rate across platforms	Ensure consistency and reproducibility of FLASH-RT data across institutions
Dedicated UHDR Treatment Planning Modules	Integrate oxygen depletion and radical kinetics into TPS algorithms	Enable biologically informed dose calculations tailored to FLASH-specific effects
Quality Assurance Frameworks	Develop UHDR-compatible phantoms and detectors; standardize QA via professional organizations (e.g., AAPM, ESTRO)	Improve reliability, safety, and clinical comparability
Mechanistic Research Advancement	Conduct multicenter preclinical studies under physiologic oxygen tensions using molecular and imaging endpoints	Elucidate biological mechanisms and identify biomarkers for FLASH effect
Preclinical Consortia Formation	Coordinate interinstitutional networks to share beam time, protocols, and data	Improve reproducibility, enable large-scale validation, and support meta-analyses
Regulatory Engagement	Collaborate with FDA/EMA to define device classes, standards, and clinical trial guidelines	Accelerate regulatory approval and ensure compliance readiness
Economic and Infrastructure Planning	Perform cost–benefit analyses; support public–private partnerships	Mitigate capital costs and promote sustainable FLASH-RT deployment

## Data Availability

Not applicable. Links are provided in the cited literature.
